# Supplementary material for: A Fluidics-Based Biosensor to Detect and Characterize Inhibition Patterns of Organophosphate to Acetylcholinesterase in Food Materials
Source: Micromachines (Basel). 2021 Apr 3;12(4):397. doi: 10.3390/mi12040397 (PMC8065683; doi:10.3390/mi12040397)
Supplement: Supplementary file 1 [file micromachines-12-00397-s001.pdf]

Article

A Fluidics-based Biosensor to Detect and Characterize Inhibition Patterns of Organophosphate to Acetylcholinesterase in Food Materials

Dang Song Pham*[1], Xuan Anh Nguyen*[1], Paul Marsh[2], Sung Sik Chu[1], Michael P.H. Lau[3], Anh H. Nguyen[2,3], Hung Cao[1,2,3]

[1] Biomedical Engineering Department, University of California Irvine, USA
[2] Electrical Engineering and Computer Science Department, University of California Irvine, USA
[3] Sensoriis, Inc. 7500 212th St SW Ste 208 Edmonds, WA, USA
* Equal contribution
¶ Co-correspondence
1 Affiliation[1]; hungcao@uci.edu
2 Affiliation[2]; hungcao@sensoriis.com
* Correspondence: Correspondence: hungcao@uci.edu; Tel: +1 (949) 824-8478

Citation: Pham, D.S.; Nguyen, X.A.; Marsh, P.; Chu, S.S.; Michael P.H. Lau3; Nguyen, A.H.; Cao, H. A Fluidics-based Biosensor to Detect and Characterize Inhibition Patterns of Organophosphate to Acetylcholinesterase in Food Materials. *Micromachines* **2021**, *12*, 397. https://doi.org/10.3390/mi12040397

Received: 4 March 2021
Accepted: 1 April 2021
Published: 3 April 2021

Publisher's Note: MDPI stays neutral with regard to jurisdictional claims in published maps and institutional affiliations.

Copyright: © 2021 by the authors. Submitted for possible open access publication under the terms and conditions of the Creative Commons Attribution (CC BY) license (http://creativecommons.org/licenses/by/4.0/).

Supporting information

Supplementary Materials: The following are available online at www.mdpi.com/xxx/s1.

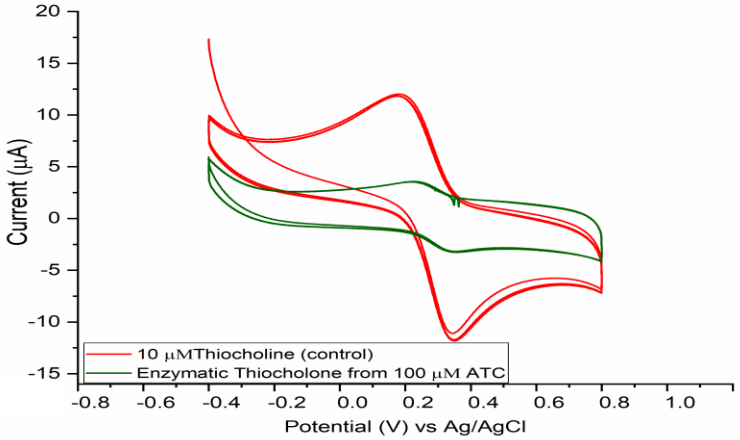

Figure S1. Cyclic voltammetric assay on composite electrode with 10 μM enzymatic thiocholine (green) and 10 μM thiocholine as a control (red).

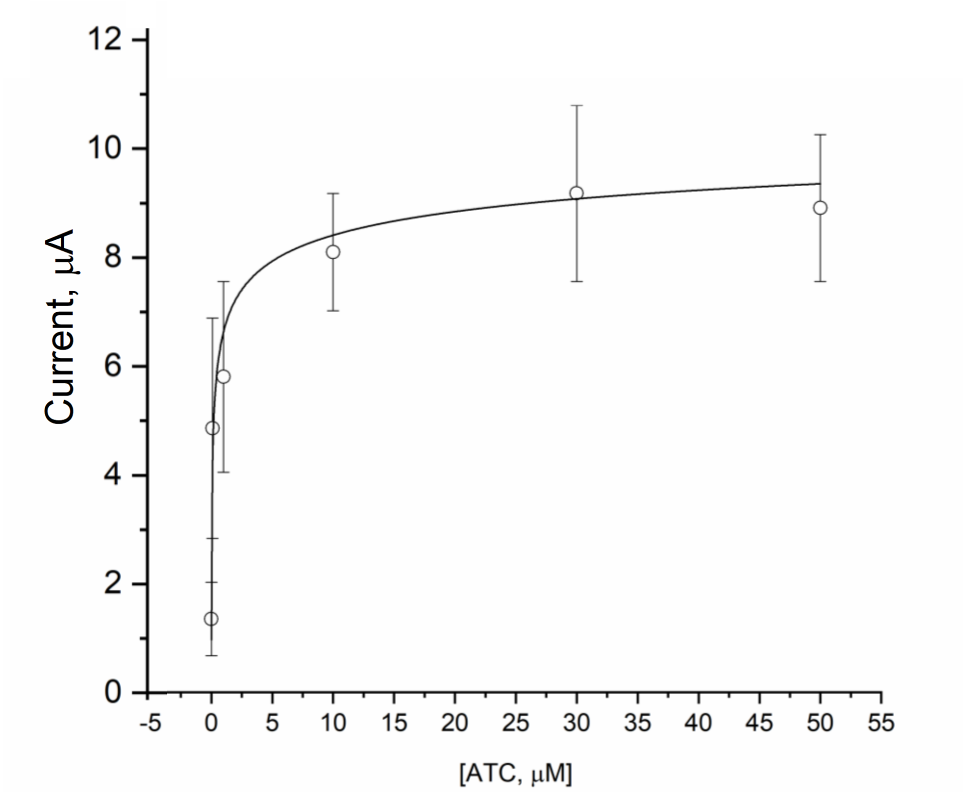

Figure S2. ATC saturation kinetics of AChE . Reaction mixture contained in 0.5 ml of different concentration of ATC as indicated and the phosphate buffer (10 mM, pH 8.0) mixed with 0.1 M KCl. Saturated current was around 30 μM of ATC.

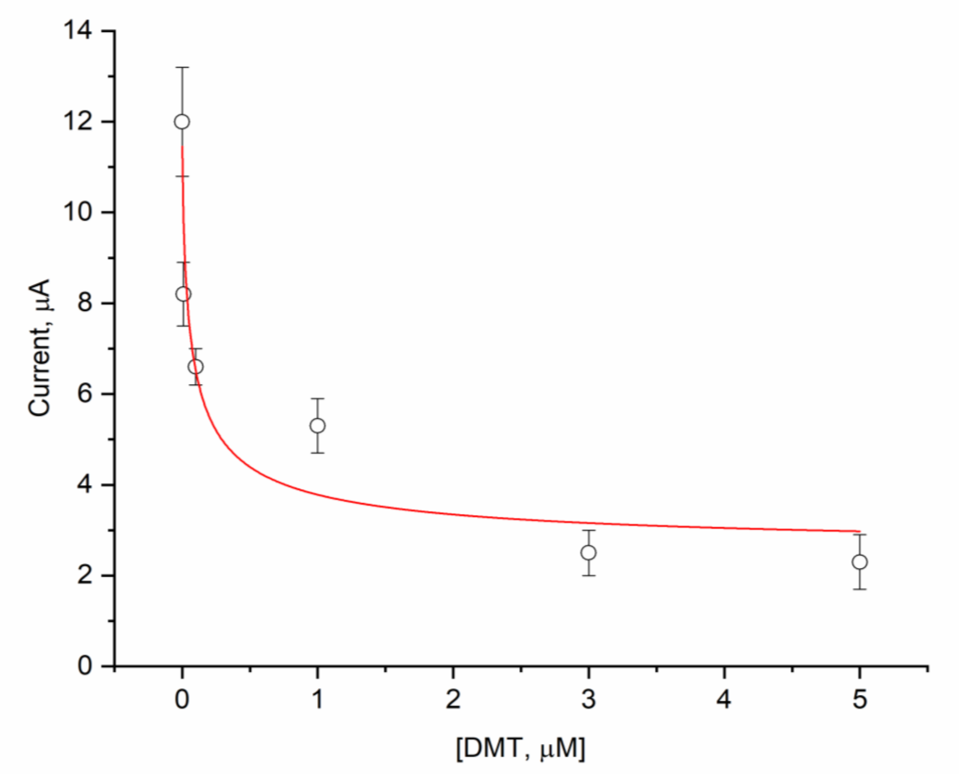

Figure S3. Concentration-based AChE inhibition. AChE activity was inhibited at 50% (IC_{50}) 0.57 μM.

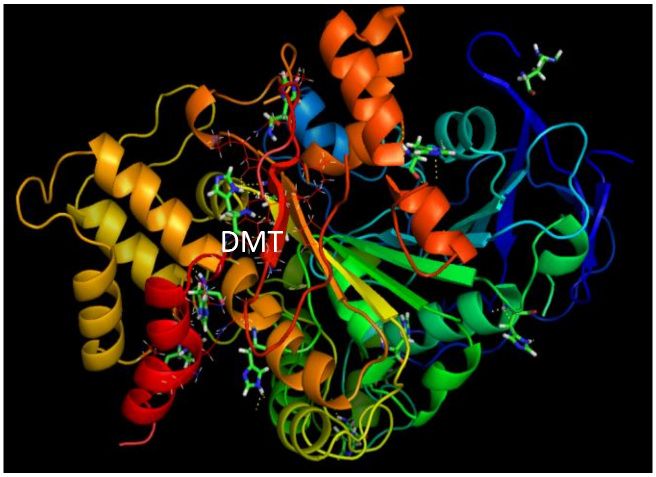

(a)

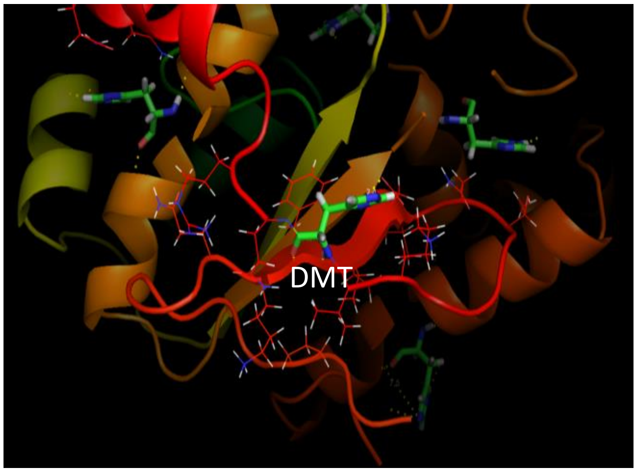

(b)

Figure S4. (a) DMT binding to another sites of AChE molecules. (b) DMT binding to active site (Ser-His-Glu). Molecular Swiss Dock[1] was used for the molecular binding modeling.

1. Grosdidier A, Zoete V, Michielin O: **SwissDock, a protein-small molecule docking web service based on EADock DSS**. *Nucleic Acids Res* 2011, **39**(Web Server issue):W270-W277.
